# Facile Preparation of Fe_3_O_4_/C Nanocomposite and Its Application for Cost-Effective and Sensitive Detection of Tryptophan

**DOI:** 10.3390/biom9060245

**Published:** 2019-06-22

**Authors:** Jun Liu, Shuai Dong, Quanguo He, Suchun Yang, Mei Xie, Peihong Deng, Yonghui Xia, Guangli Li

**Affiliations:** 1 Hunan Key Laboratory of Biomedical Nanomaterials and Devices, College of Life Sciences and Chemistry, Hunan University of Technology, Zhuzhou 412007, China; liu.jun.1015@163.com (J.L.); 15674180029@163.com (S.D.); hequanguo@126.com (Q.H.); scyang2019@163.com (S.Y.); meixie1015@126.com (M.X.); 2 Key Laboratory of Functional Metal-Organic Compounds of Hunan Province; Key Laboratory of functional Organometallic Materials of Hunan Provincial Universities; Department of Chemistry and Material Science, Hengyang Normal University, Hengyang 421008, China; 3 Zhuzhou Institute for Food and Drug Control, Zhuzhou 412000, China; Sunnyxia0710@163.com

**Keywords:** Fe_3_O_4_/C composite, tryptophan, voltammetric detection, second derivative linear scan voltammetry

## Abstract

In this study, we reported facile synthesis of Fe_3_O_4_/C composite and its application for the cost-effective and sensitive determination of tryptophan (Trp) in human serum samples. Fe_3_O_4_/C composites were prepared by a simple one-pot hydrothermal method followed by a mild calcination procedure, using FeCl_3_∙6H_2_O as Fe_3_O_4_ precursor, and glucose as reducing agent and carbon source simultaneously. The Fe_3_O_4_/C composite modified glassy carbon electrode (Fe_3_O_4_/C/GCE) was prepared by drop-casting method. The microstructure and morphology of Fe_3_O_4_/C composite was characterized by powder X-ray diffraction (XRD) and scanning electron microscopy (SEM), respectively. Due to large specific surface area and synergistic effect from Fe_3_O_4_ nanoparticles and carbon coating, Fe_3_O_4_/C composite showed excellent electrocatalytic activity toward the oxidation of Trp. As a result, the proposed Fe_3_O_4_/C/GCE displayed superior analytical performances toward Trp determination, with two wide detection ranges (1.0–80 μM and 80–800 μM) and a low detection limit (0.26 μM, S/N = 3). Moreover, successful detection of Trp in human serum samples further validate the practicability of the proposed sensor.

## 1. Introduction

Tryptophan (Trp), one of eight essential amino acids in human body, plays indispensable roles in maintaining human growth, metabolism, and positive nitrogen balance [1]. Trp is also a vital precursor for melatonin and serotonin. It has been reported that metabolic disorders or abnormal levels of Trp may cause several serious diseases including Alzheimer’s diseases, delusions, and hallucinations [2,3]. Even worse, highly oxidized products of Trp can even lead to some horrible cancers [3]. Trp has become a most promising biomarkers for early diagnosis of some cancers, such as gastric cancer and breast carcinoma. For instance, early diagnosis of gastric cancer can be realized by detecting Trp level in gastric fluid [4]. Hence, it is particularly urgent to develop rapid and reliable techniques to detect Trp in physiological samples. 

At present, various analytical techniques have been developed for the identification and determination of Trp, including but not limited to high-performance liquid chromatography [4], capillary electrophoresis [5], spectrofluorimetry [6], colorimetric method [7], chemiluminescence [8], and electroanalytical methods [3,9,10,11,12,13,14,15,16,17,18,19,20,21,22,23,24]. Compared with other techniques, electroanalytical methods are very suitable for the determination of biomolecules due to their considerable advantages, such as rapid response, facile operation, high sensitivity, and easy miniaturization. Moreover, Trp has desirable electrochemical activity, which is favorable for electrochemical determination [9,10,11,12,13,14,15,16,17,18,19,20,21,22,23,24]. However, it is difficult to directly detect Trp on bare electrodes, owing to the high overpotential resulting from the sluggish electrode kinetics [3]. The most popular solution is to develop highly sensitive modified electrodes. Various modified electrodes have been successfully applied to detect Trp in actual samples [3,9,10,11,12,13,14,15,16,17,18,19,20,21,22,23,24]. Nevertheless, they still suffer from some draws, including complexity [3,21,22,23], low detection limit (LOD), narrow linear range [9,10,11,12,13,14,15,16,21,23,24], and the high cost of precious metal nanoparticles [3,17,18,19,20], which severely hinder their widespread practice applications. Therefore, the development of novel low-cost, but highly efficient, modification materials for sensing Trp is utmost critical and remains a great challenge. 

To date, various advanced nanostructures have been developed as sensitive sensing materials for the construction of electrochemical sensors [25,26,27,28,29]. It is well-known that transition metal oxide nanomaterials have obvious advantages over noble metal nanoparticles in terms of the cost and abundance [30,31,32,33]. Nanostructured transition metal oxides such as TiO_2_ [15,16], SnO_2_ [9], CuO [11], and Ta_2_O_5_ [34] have been utilized for the detection of Trp. Despite the wide variety of transition metal oxides available, Fe_3_O_4_ nanoparticles (Fe_3_O_4_ NPs) are highly encouraged as sensing materials because of their low fabrication cost, environmental friendliness, and excellent electrochemical properties [35]. Moreover, the good biocompatible nature of Fe_3_O_4_ NPs endows them attractive candidates for various biosensors [36]. For example, highly ordered mesoporous Fe_3_O_4_ NPs with high surface area showed high catalytic activity toward dopamine, with wide linear detection range of 2–600 nM and low detection limit of 0.8 nM [37]. Magnetic Fe_3_O_4_ NPs modified electrode showed excellent catalytic activity toward the detection of Sundan I, with linear detection range of 1–20 μM and low LOD of 0.001 μM [38]. However, the poor electrical conductivity of Fe_3_O_4_ NPs has restricted their applications on electrochemical sensors. To address this issue, coupling of Fe_3_O_4_ NPs with highly conductive carbonaceous materials is very effective to increase electrical conductivity, and thus, enhance electrochemical sensing properties [39]. More recently, coupling of Fe_3_O_4_ with carbon nanofibers and multiwalled carbon nanotubes has been proven to be very feasible for improving the electrical conductivity [40,41]. In our recent studies, compositing Fe_3_O_4_ with graphene nanosheets showed low charge resistant (*R*_ct_) and superior electrochemical sensing performances toward the sensitive detection of bioactive molecules, such as dopamine and rutin [42,43]. However, the tedious route and high cost for the synthesis of carbonaceous materials (i.e., carbon nanotubes, carbon nanofibers, graphene) will undoubtedly increase the overall production cost of the sensor [44,45], which adversely affects their commercial applications. Therefore, compositing with carbonaceous materials that possess merits of facile preparation and high electrical conductivity not only significantly improves the sensitivity, but also greatly decreases the fabrication cost.

Herein, a facile one-pot route followed by a mild calcination procedure has been proposed to prepare Fe_3_O_4_/C composite for the sensitive detection of Trp, using FeCl_3_∙6H_2_O as Fe_3_O_4_ precursor, and glucose as reducing agent and carbon source simultaneously. Fe_3_O_4_/C composites not only retain unique properties from individual component, but yield synergistic enhancement effect toward the oxidation of Trp. As a result, the electrochemical response of Trp were boosted greatly. The voltammetric parameters, such as solution pH, accumulation potential, and accumulation time, were systematically optimized. At last, the proposed sensor was successfully applied to detect Trp from human serum samples with good recovery. 

## 2. Materials and Methods 

### 2.1. Reagents and Solution

L-tryptophan (Trp) was purchased from Aladdin Bio-Chem Technology Co., LTD (Shanghai, China). Human serum samples were taken from People’s Hospital of Zhuzhou. Ferric trichloride hexahydrate (FeCl_3_∙6H_2_O), urea (CH_4_N_2_O), glucose (C_6_H_12_O_6_), α-Al_2_O_3_ slurry (particle size: 0.3 μm and 0.05 μm), disodium phosphate dodecahydrate (Na_2_HPO_4_), sodium dihydrogen phosphate (NaH_2_PO_4_∙12H_2_O), potassium nitrate (KNO_3_), potassium ferricyanide (K_3_[Fe(CN)_6_]), potassium ferrocyanide (K_4_[Fe(CN)_6_]), and ethyl alcohol were supplied by Sinopharm Chemical Reagent Co., Ltd. (Shanghai, China). All these chemicals were used as received without further treatment. A series of different concentrations of Trp standard solutions was prepared by appropriately diluting 1 mM stock solution of Trp with 0.1 M phosphate buffer saline solution (PBS, pH 6.5). Ultrapure water (18.2 MΩ) was used throughout the experiments.

### 2.2. Synthesis of Fe_3_O_4_/C Composites 

Fe_3_O_4_/C composite material was synthesized via a facile one-pot hydrothermal method. Typically, 3.6 g of glucose, 0.5 g of FeCl_3_∙6H_2_O, and 11.6 g of urea were dissolved in 80 mL of ultrapure water under stirring for 10 min. Then, the mixture solution was transferred to a 100 mL Teflon-lined stainless-steel autoclave and reacted at 180 °C for 14 h. After it was cooled down to room temperature, the product was separated by external magnetic field. Then, the product was washed by ultrapure water/ethanol for several times and dried in vacuum oven for 12 h. Finally, the Fe_3_O_4_/C was obtained after a mild calcination at 600 °C for 4 h under Ar atmosphere protection. For comparison, the pure Fe_3_O_4_ NPs were prepared by the same method in absence of glucose.

### 2.3. Fabrication of Fe_3_O_4_/C Modified Electrode

At first, the glassy carbon electrodes (GCEs) were polished by α-Al_2_O_3_ slurry with different fine sizes (0.3 μm and 0.05 μm). Then, they were alternately washed by ethyl alcohol and ultrapure water under ultrasonication for 2~3 times (each for 1 min), respectively. Five microliters of Fe_3_O_4_/C dispersion (1 mg/mL) was drop-casted onto the surface of polished GCE, then dried under an infrared lamp to obtain Fe_3_O_4_/C composites modified GCE (Fe_3_O_4_/C/GCE). For comparison, pure Fe_3_O_4_ NPs modified GCE (Fe_3_O_4_/GCE) was also prepared by the similar method. 

### 2.4. Material Characterization

Scanning electron microscopy (SEM) images were collected from a Hitachi S4800 (Hitachi, Tokyo, Japan) operating at 5 kV. Powder X-ray diffraction (XRD) patterns were recorded on an X-ray diffractometer (PANalytical, Almelo, Holland) operating at 40 kV and 40 mA with Cu Kα radiation (λ = 0.1542 nm).

### 2.5. Electrochemical Experiments

The electrochemical behaviors of as-prepared Fe_3_O_4_/C/GCE were investigated in a freshly prepared 0.1 M PBS containing 10 μM Trp by cyclic voltammetry (CV). The quantitative analysis of Trp were performed by second derivative linear scan voltammetry (SDLSV). CV and SDLSV were recorded on an electrochemical workstation (CHI 760E, Shanghai Chenhua Inc., Shanghai, China) and a Polarographic Analyzer (JP-303E, Chengdu Instrument Company, Chengdu, China), respectively. Both CVs and SDLSVs were measured using a conventional three-electrode assemble, in which bare or modified GCEs worked as working electrode, while platinum wire electrode and saturated calomel electrode (SCE) acted as counter electrode and reference electrode, respectively. Before all the electrochemical measurements for Trp, a suitable accumulation was performed under stirring at 500 rpm to increase the electrochemical responses. After a 5 s rest, both the CVs and SDLSVs were measured at a scanning rate of 0.1 V/s. The potential was scanned from −0.2 to 1.2 V for both CV and SDLSV.

### 2.6. Detection of Trp in Human Serum

Under the optimal voltammetric conditions, Trp in human serum was detected using SDLSV technique. Typically, 1 mL human serum sample was diluted to 10 mL with 0.1 M PBS (pH 6.5). Standard addition method was further used to validate the reliability of the proposed sensor. 

## 3. Results and Discussion

### 3.1. Microstructure and Morphology Characterization of Fe_3_O_4_/C Composites

**Figure 1:**
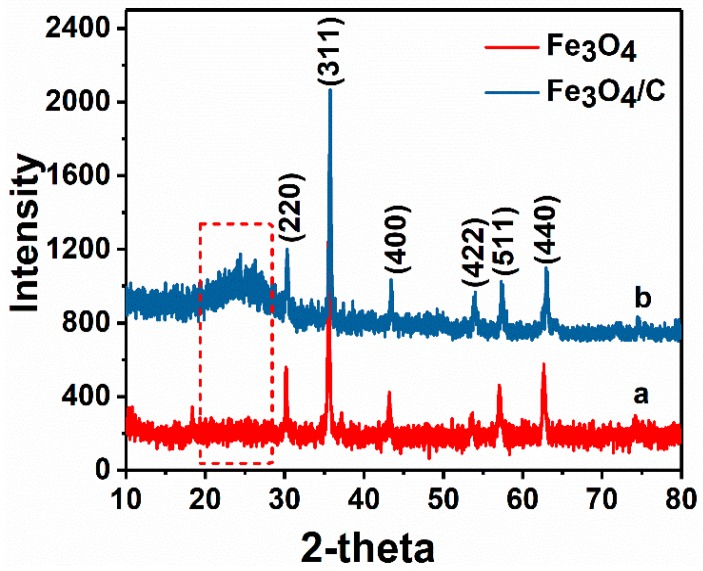
XRD patterns of pure Fe_3_O_4_ NPs (curve a) and Fe_3_O_4_/C composite (curve b).

The microstructures of pure Fe_3_O_4_ NPs and Fe_3_O_4_/C composite were characterized by power X-ray diffraction (XRD) technique. As presented in Figure 1, curve a and curve b are the XRD patterns of pure Fe_3_O_4_ NPs and Fe_3_O_4_/C composite, respectively. The emerged various sharp peaks indicating that these as-prepared samples show good crystallization performance. Moreover, the diffraction peaks located at 30.063°, 35.451°, 43.037°, 53.546°, 57.166°, and 62.726° are corresponding to crystal facets of (220), (311), (400), (422), (511), and (440), respectively. This result is in good accordance with standard PDF card of magnetite (JSPDS 01-1111, α = 8.374Å). The corresponding peaks of magnetite in the Fe_3_O_4_/C sample are also apparent, indicating that a good crystallization is observed. Moreover, the crystallization is not influenced by carbon coating. Besides, a wide peak located at 20°~30° is observed, which could be ascribed to diffraction peak of amorphous carbon layer. These results confirm that the Fe_3_O_4_/C nanocomposite was synthesized successfully.

**Figure 2:**
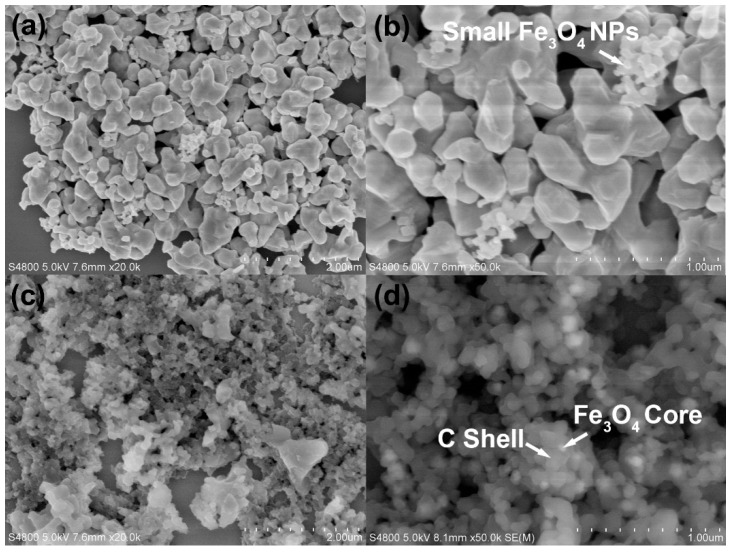
SEM images of pure Fe_3_O_4_ nanoparticles (**a**,**b**) and Fe_3_O_4_/C composite nanoparticles (**c**,**d**).

The surface morphologies of as-prepared pure Fe_3_O_4_ NPs and Fe_3_O_4_/C nanocomposites were further characterized by scanning electron microscopy (SEM). As presented in Figure 2a,b, the irregular shapes of Fe_3_O_4_ NPs are observed with excellent dispersity, and the size of these Fe_3_O_4_ NPs is roughly estimated as 200~300 nm. However, in the Fe_3_O_4_/C composite sample, the particle size is larger than that of pure Fe_3_O_4_ NPs (Figure 2c,d). Because of the carbon coating shell, the nanoparticles are aggregated with each other. Interestingly, the contrast of carbon shell and Fe_3_O_4_ core is manifested in Figure 2b, because the electron bombardment under high voltage propels the electron transmission. The light spots could be considered as Fe_3_O_4_ nanoparticles because of heavy atom Fe, and other dark parts are identified as carbon shell. It is a side evidence that the core-shell Fe_3_O_4_/C composite nanoparticles are formed successfully.

### 3.2. Electrochemical Active Area 

**Figure 3:**
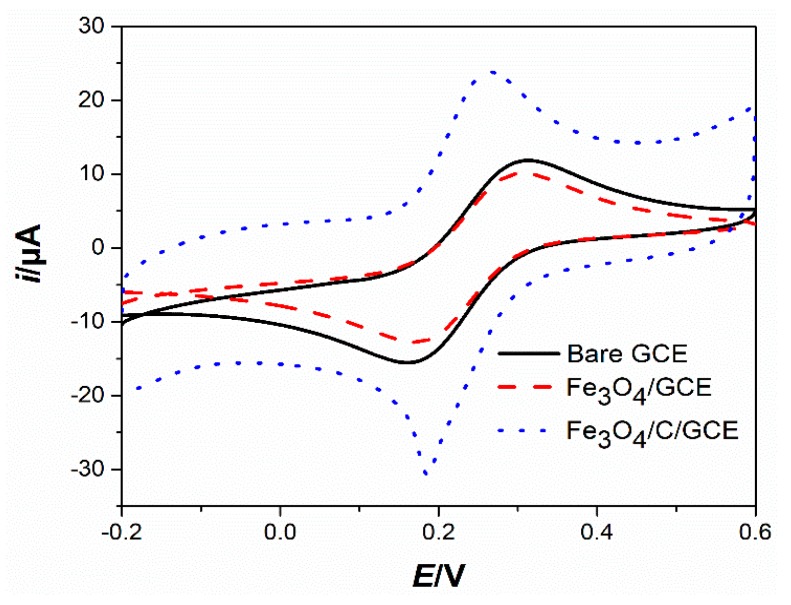
Electrochemical active areas of different electrodes were investigated by CV in 0.1 M PBS containing 0.5 mM of K_3_[Fe(CN)_6_].

The CVs of bare GCE, Fe_3_O_4_/GCE and Fe_3_O_4_/C/GCE were recorded in 0.1 M PBS containing 0.5 mM K_3_Fe(CN)_6_ (Figure 3). A pair of quasi-reversible redox peaks (*i*_pa_ = 13.69 μA; *i*_pc_ = 14.71 μA) are observed at the bare GCE, with peak-peak separation (∆*E*_p_) of 0.153 V. At the Fe_3_O_4_/GCE, the redox peak currents (*i*_pa_ = 12.03 μA; *i*_pc_ = 12.61 μA) are inferior to those of bare GCE, probably because of the poor electrical conductivity of Fe_3_O_4_ NPs. Moreover, the peak-peak separation (∆*E*_p_) decrease to 0.145 V, suggesting Fe_3_O_4_ NPs effectively reduced the active polarization. Among these three electrodes, Fe_3_O_4_/C/GCE shows the largest response peak currents (*i*_pa_ = 19.04 μA; *i*_pc_ = 25.29 μA) with lowest peak-peak potential separation (∆*E*_p_ = 0.079 V), due to the synergistic effect from high electrocatalytic activity of Fe_3_O_4_ NPs and excellent electrical conductivity of carbon shells. The cathodic peak currents (*i*_pc_) of bare GCE, Fe_3_O_4_/GCE, and Fe_3_O_4_/C/GCE are 14.71 μA, 12.61 μA, and 25.29 μA, respectively. Referring to the *Randles-Sevcik* equation [46,47,48]:

*i*_pc_ = 2.691 × 10^5^*n*^3/2^*D*^1/2^*ν*^1/2^*A C* (1)

where *i*_pc_ represents the cathodic peak currents of K_3_[Fe(CN)_6_], *n* represents the transferred electron number during the redox reaction of K_3_[Fe(CN)_6_], *D* is the diffusion coefficient of K_3_[Fe(CN)_6_] (7.6 × 10^−6^ cm^2^ s^−1^ [49]), *v* is the scanning rate (V s^−1^), *A* is the electrochemical active area (cm^2^), and *C* is the concentration of K_3_[Fe(CN)_6_] (mol cm^−3^). The electrochemical active areas of bare GCE, Fe_3_O_4_/GCE and Fe_3_O_4_/C/GCE are calculated as 0.01255 cm^2^, 0.01075 cm^2^, and 0.02209 cm^2^, respectively. The electrochemical active area of Fe_3_O_4_/C/GCE is about two-fold that of bare GCE, indicting that the Fe_3_O_4_/C composite modification could enhance the effective electroactive surface area, which can facilate the adsorption of target analytes on the electrode surface and offer more catalytic active sites, and thus accelerate the redox process of target analytes. 

### 3.3. Electrochemical Responses of Trp on Various Modified Electrodes

**Figure 4:**
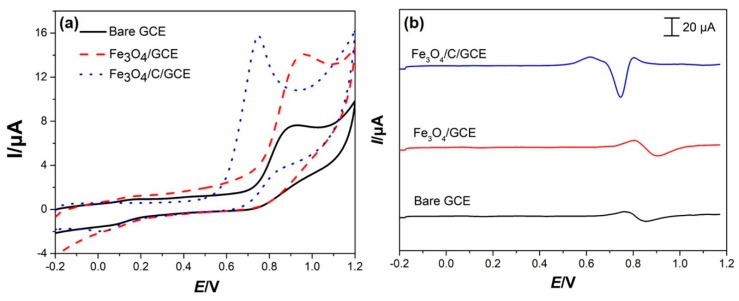
CV (**a**) and second derivative linear scan voltammetry (SDLSV) (**b**) responses of 10 μM Trp on bare GCE, Fe_3_O_4_/GCE, and Fe_3_O_4_/C/GCE. Supporting electrolyte: 0.1 M PBS (pH 6.5); Scanning rate: 0.1 V/s.

The electrochemical responses of 10 μM Trp on bare GCE, Fe_3_O_4_/GCE, and Fe_3_O_4_/C/GCE electrodes were investigated by CV (Figure 4a). Clearly, cathodic peaks are not observed at all electrodes, implying the oxidation of Trp is an irreversible process. At bare GCE, a relatively poor anodic peak (*i*_pa_ = 4.603 μA) is observed at 0.929 V. After the modification of GCE with Fe_3_O_4_ NPs, the *i*_pa_ of Trp increases to 7.414 μA, suggesting Fe_3_O_4_ NPs have high electrocatalytic activity toward Trp oxidation. However, the anodic peak shifts positively to 0.963 V, due to the poor electrical conductivity of Fe_3_O_4_ NPs. When decorated with Fe_3_O_4_/C nanocomposites, a sharp anodic peak appears with highest response peak current (13.13 μA) and lowest working potential (0.750 V). The *i*_pa_ of 10 μM Trp at Fe_3_O_4_/C/GCE is about two times higher than that at bare GCE. The enhanced anodic peak current and decreased anodic peak potential is mainly due to the synergistic effect of Fe_3_O_4_ NPs and carbon coating. Specifically, Fe_3_O_4_ NPs offer favorable catalytic active sites for the Trp oxidation; The carbon coating improves the electrical conductivity and decreases the charge transfer resistance (*R*_ct_). Besides, the large electrochemical active area of Fe_3_O_4_/C composite also contributed to the enhancement on the *i*_pa_ of Trp. The SDLSV responses of 10 μM Trp at various electrodes are shown in Figure 4b. On bare GCE, a weak anodic peak (*i*_pa_ = 9.852 μA) occurs at 0.852 V. When modified with Fe_3_O_4_ NPs, the anodic peak shifts to 0.901 V and the anodic peak current increases to 16.42 μA. After the modification of GCE with Fe_3_O_4_/C composites, a well-shaped anodic peak was observed with largest anodic peak current of 46.55 μA and lowest anodic peak potential of 0.746. The SDLSV results are highly consistent with the CV results. Obviously, SDLSV is more sensitive than CV for Trp detection. So, SDLSV was performed for the detection of Trp.

### 3.4. Optimization of Voltammetric Conditions

#### 3.4.1. Effect of Solution pH

**Figure 5:**
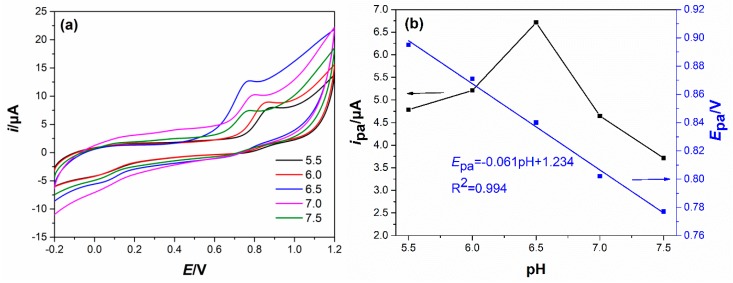
Effect of solution pH on the electrochemical responses of 10 μM Trp. (**a**) CV curves in different pH PBS. (**b**) Effect of solution pH on the anodic peak currents (black line) and the anodic peak potentials (blue line). Supporting electrolyte: 0.1M PBS; Scanning rate: 0.1 V/s; Accumulation potential: 0.2 V; Accumulation time: 210 s.

In order to investigate the influence of solution pH, the CVs of 10 μM Trp were measured in various pH PBS (Figure 5a). As plotted in Figure 5b, the *i*_pa_ increases with pH varying from 5.5 to 6.5, then gradually declines with pH further increasing. So, pH 6.5 was selected for the subsequent measurements. Moreover, the anodic peak potential (*E*_pa_) is highly linear to solution pH, and the corresponding linear regression equation is *E*_pa_(V) = −0.061pH + 1.234 with correlation coefficient (R^2^) of 0.994. The slope (−61mV/pH) is very close to theoretical value from Nernst equation (−59 mV/pH), suggesting the equal number of protons (H^+^) and electrons (e^−^) are involved in the electrochemical oxidation of Trp [50]. 

#### 3.4.2. Effect of Scanning Rate

**Figure 6:**
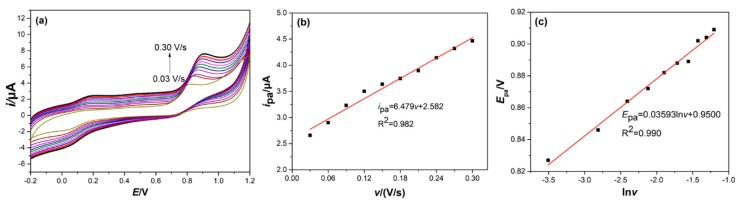
Effect of scanning rate (*v*) on the electrochemical responses of 10 μM Trp. (**a**) CVs curves of 10 μM Trp on Fe_3_O_4_/C/GCE recorded at various scanning rates (*v*); (**b**) Linear relationship between the anodic peak currents (*i*_pa_) and scanning rates (*v*); (**c**) Linear relationship between the anodic peak potentials (*E*_pa_) and Napierian logarithm of scanning rates (ln*v*).

**Scheme 1:**
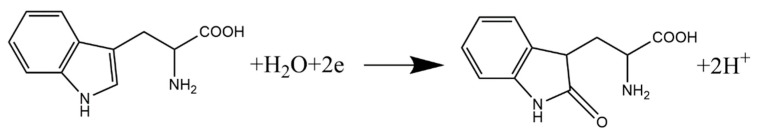
Schematic diagram of the electrochemical mechanism of Trp oxidation.

The CV curves of 10 μM Trp were recorded at different scanning rates (0.03~0.30 V s^−1^) in 0.1 M PBS (pH 6.5), aiming to reveal the electrooxidation mechanism of Trp on Fe_3_O_4_/C/GCE. As can be seen from Figure 6a, the *i*_pa_ of Trp increases with the speeding of scanning rates, but the background currents also increase with the increase of the scanning rates. More importantly, a good linear relationship between anodic peak currents (*i*_pa_) and scanning rates (ln*v*) is found in Figure 6b. The linear equation can be expressed as *i_pa_* (μA) = 6.479*v* (V/s) + 2.582 (R^2^ = 0.982). It demonstrates the electrooxidation of Trp on Fe_3_O_4_/C/GCE is an adsorption-controlled process [30,31,33]. As shown in Figure 6c, the anodic peaks are shifted toward the positive potential direct. The anodic peak potentials linearly increase with Napierian logarithm of scanning rates (ln*v*), and the linear equation can be expressed as *E*_pa_(V) = 0.03593ln*v* + 0.9500 (R^2^ = 0.990). As for an adsorption-controlled and totally irreversible electrode process, it should follow the Lavrion’s theory as follows [51]:

(2) $$E_{p}=E^{0}+\frac{RT}{\alpha nF}\ln\frac{{RTk}^{0}}{\alpha nF}+\frac{RT}{\alpha nF}\ln v$$

where *E*_p_ denotes the peak potential, E^0^ denotes the formal potential (V), *R* denotes the gas constant (8.314 J K^−1^ mol^−1^), *T* denotes the temperature (298.15 K), α denotes the charge transfer coefficient, *n* represents the electron transferred number, *F* is the Faraday constant (96, 485 C mol^−1^), *k*^0^ represents standard heterogeneous transfer rate (s^−^^1^), and *v* is the scanning rate (V/s), respectively. Referring to Laviron’s equation, the slope of the linear equation is equivalent to RT/αnF (where α is assumed to be 0.5 for a totally irreversible electrode process [52]). Hence, *n* is calculated to be around 2. The dependence of solution pH on the anodic peak potential (mentioned in Section 3.4.1) confirms that the equal number of protons (H^+^) and electrons (e^−^) participate in the electrochemical oxidation of Trp. So, the electrooxidation of Trp at Fe_3_O_4_/C/GCE is a two-electrons and two-protons process (2e^−^, 2H^+^). The electrochemical mechanism of Trp oxidation is described in Scheme 1, which is in good agreement with previous reports [13,53,54].

#### 3.4.3. Effect of Accumulation Parameters 

**Figure 7:**
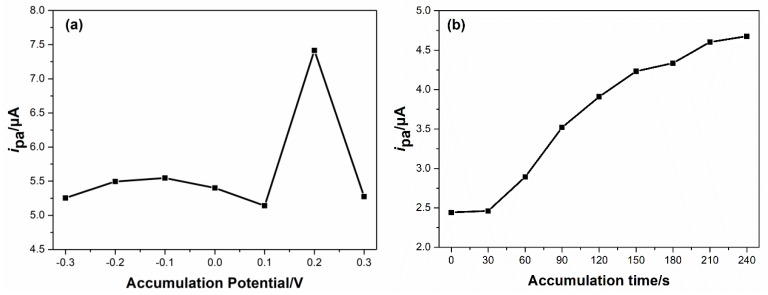
Effect of accumulation potential (**a**) and accumulation time (**b**) on the anodic peak currents of 10 μM Trp.

Accumulation is very feasible to improve the sensitivity of the proposed sensor, since the electrochemical oxidation of Trp on the Fe_3_O_4_/C/GCE is an adsorption-controlled process. The Trp was accumulated on the surface of Fe_3_O_4_/C/GCE at various potentials for 240 s, then their *i*_pa_ of were measured by SDLSV. As presented in Figure 7a, the maximum *i*_pa_ is achieved at 0.2 V. Hence, 0.2 V was recommended as the optimal accumulation potential. Moreover, the influence of accumulation time on the *i*_pa_ were also investigated at fixed accumulation potential of 0.2 V (Figure 7b). As the accumulation time prolongs, the *i*_pa_ gradually increase during the first 210 s, then remain stable with further increasing accumulation time, suggesting the saturation adsorption of Trp. Hence, the accumulation was performed at 0.2 V for 210 s in the following measurements.

### 3.5. Calibration Curves, Linear Response Ranges and Limit of Detection

**Figure 8:**
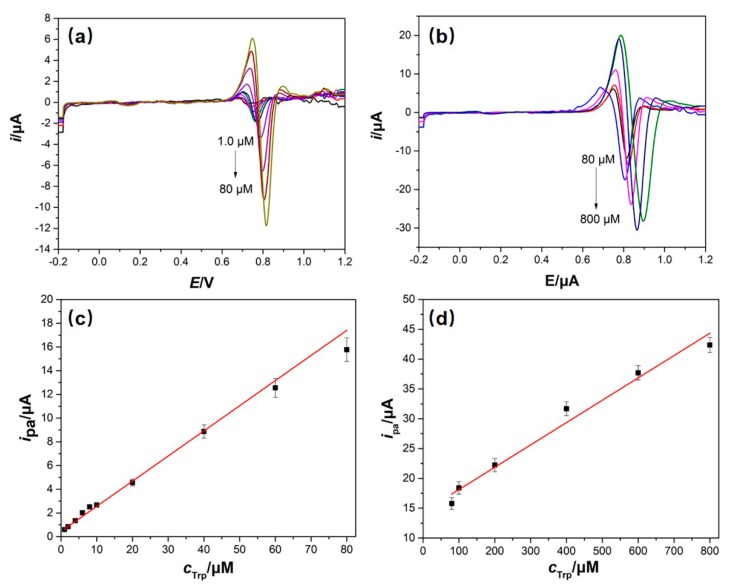
curves of Trp with the concentrations ranging from 1 μM to 80 μM (**a**) and from 80 μM to 800 μM (**b**); Calibration curves between the anodic peak currents (*i*_pa_) and Trp concentrations (*c*_Trp_) in the range of 1–80 μM (**c**) and 80–800 μM (**d**). Supporting electrolyte: 0.1M PBS (pH 6.5); Scanning rate: 0.1 V/s; Accumulation potential: 0.2 V; Accumulation time: 210 s.

SDLSV curves of various concentrations of Trp are shown in Figure 8a,b. As can be seen, the response *i*_pa_ increase with Trp concentration increasing. Moreover, obvious anodic peak shifts are observed at higher concentration regions, because the concentration polarization lead to positive shift of anodic peak currents. Notably, there are two linear response regions, namely 1.0–80 μM (Figure 8c) and 80–800 μM (Figure 8d). The corresponding linear regression equations are *i*_pa_ (μA) = 0.2120*c* (μM) + 0.4406 (R^2^ = 0.986) and *i*_pa_ (μA) = 0.03746*c* (μM) + 14.37 (R^2^ = 0.979), respectively. The slopes of two linear regions are very different, because the contributions of adsorptive and diffusion current components are changed in 3~4 orders within the concentration range. The LOD was estimated according to the equation: LOD = 3σ/s, where σ is the standard deviation of the blank solution and *s* is the slope of the calibration plots. The LOD (S/N = 3) is estimated as 0.26 μM. The superb sensing performance is mainly due to synergistic effect from high catalytic activity of Fe_3_O_4_ NPs and excellent conductive carbon coating. A comparison of analytical performances between our proposed sensor and the existing electrochemical sensors is summarized in Table 1. To our surprise, the sensing properties of our proposed sensor are at least comparable to or even superior than previous reports [9,10,11,12,13,14,15,16,21,23,24,34]. Moreover, Fe_3_O_4_/C/GCE has outstanding advantages including facile preparation and low costs, which is very important for the practical applications. 

### 3.6. Repeatability, Reproducibility and Stability Assays

**Figure 9:**
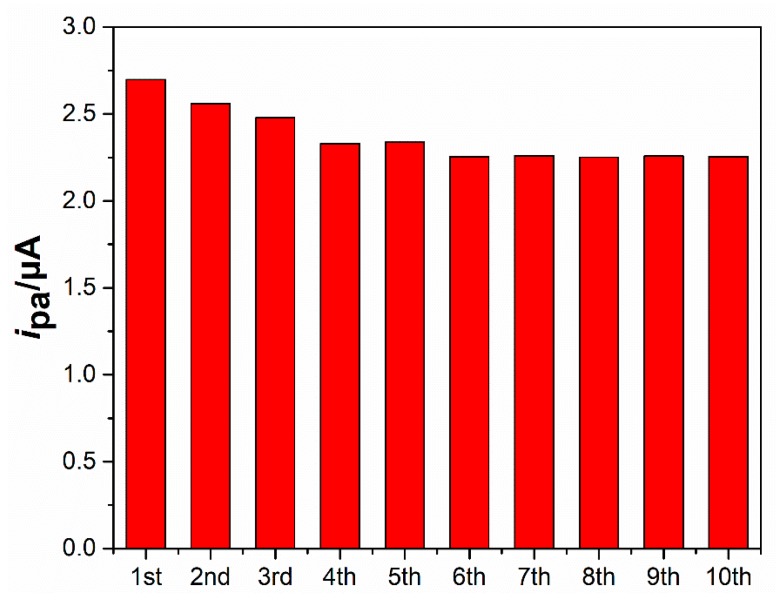
Response anodic peak currents of 10 μM Trp for ten successive measurements (*n* = 10).

The repeatability and reproducibility assays were also carried out to verify the practicability. The repeatability was examined by ten successive measurements of the i_pa_ of 10 μM Trp (Figure 9). The relative standard deviation (RSD) of 5.65% (*n* = 10), demonstrating good repeatability. The reproducibility was also checked by paralleled measurements of 10 μM Trp, using five Fe_3_O_4_/C/GCEs fabricated by the same procedures. The RSD (4.78%, *n* = 5) is accepted, suggesting highly reproducible for electrode preparation. Finally, the stability was assessed by monitoring the anodic peak currents of 10 μM Trp at a same Fe_3_O_4_/C/GCE during a week. When not in use, Fe_3_O_4_/C/GCE was immersed in Trp solution and stored at a 4 °C refrigerator. After a week, the anodic peak current retains 90.4% of the original response. 

### 3.7. Detection Trp in Human Serum Samples

SDLSV was adopted for quantitative analysis of Trp due to its superior sensitivity and high resolution [30,31,33]. The detection results were listed in Table 2. The Trp concentration in human serum sample is 20.13 μM with RSD of 1.5% (*n* = 3). Moreover, the good recoveries (105–107%) shows the proposed Fe_3_O_4_/C/GCE has great application prospects in the detection of Trp from various real samples.

## 4. Conclusions

In this study, we reported facile one-pot synthesis of Fe_3_O_4_/C composite and its application for cost-effective and sensitive detection of tryptophan (Trp) in human serum samples. The Fe_3_O_4_/C/GCE was prepared by drop-casting method. Owing to the large electrochemical active area and synergistic effect from Fe_3_O_4_ NPs and carbon coating, the response anodic peak current enhanced greatly. As a result, the proposed Fe_3_O_4_/C/GCE showed two wide linear detection ranges (1.0–80 μM and 80–800 μM) and a low LOD of 0.26 μM. Finally, the proposed Fe_3_O_4_/C/GCE successfully realized the detection of Trp in human serum samples with satisfactory recoveries (105–107%). Together with facile preparation and low cost, Fe_3_O_4_/C/GCE has shown tremendous application prospects on the detection of Trp from various actual samples.

## Figures and Tables

**Table 1:** Comparison of the sensing performances toward Trp on different modified electrodes.

Electrode	Technique	Linear Range (μM)	LOD (μM)	Ref
TBABr-βCD/MWCNTs/GCE	DPV	1.5~30.5	0.07	[21]
NiO–CuO/graphene/GCE	SWV	0.3~40	0.1	[11]
β-CD/carbon QDs/GCE	DPV	5~270	0.16	[12]
Nafion/TiO_2_-graphene/GCE	DPV	5.0~140	0.7	[16]
PAA-MWCNTs/GCE	DPV	1~500	0.81	[23]
TiO_2_-graphene/4-ABSA/GCE	DPV	5~200	0.3	[15]
4FEPEM/CPE	DPV	0.85~63.4	0.56	[14]
Butyrylcholine/GCE	DPV	2~60.0	0.6	[13]
Cobalt Salophen/CNTPE	DPV	0.5~50.0	0.7	[24]
Ta_2_O_5_/rGO/GCE	SDLSV	1~8; 8~80; 80~800	0.84	[34]
Nanoporous carbon/GCE	Amperometry	1~103	0.03	[10]
RGO/SnO_2_/GCE	DPV	1~100	0.04	[9]
Fe_3_O_4_/C/GCE	SDLSV	1~80; 80~800	0.26	This work

**Table 2:** Detection of Trp in human serum sample by SDLSV (*n* = 3).

Sample	Detected (μM)	RSD (%)	Added (μM)	Total Found (μM)	RSD (%)	Recovery (%)
No. 1	20.13	1.50	20.00	41.30	2.50	105%
No. 1	20.13	1.50	40.00	63.00	1.80	107%
